# Word learning as category formation

**DOI:** 10.1371/journal.pone.0327615

**Published:** 2025-07-03

**Authors:** Spencer Caplan

**Affiliations:** Linguistics Program, CUNY Graduate Center, New York, New York, United States of America; University of Padova, ITALY

## Abstract

A fundamental question in word learning is how, given only evidence about what objects a word has previously referred to, children are able to generalize to the correct class. How does a learner end up knowing that “poodle” only picks out a specific subset of dogs rather than the broader class and vice versa? Numerous phenomena have been identified in guiding learner behavior such as the “suspicious coincidence effect” (SCE)—that an increase in the sample size of training objects facilitates more narrow (subordinate) word meanings. While SCE seems to support a class of models based in statistical inference, such rational behavior is, in fact, consistent with a range of algorithmic processes. Notably, the broadness of semantic generalizations is further affected by the temporal manner in which objects are presented—either simultaneously or sequentially. First, I evaluate the experimental evidence on the factors influencing generalization in word learning. A reanalysis of existing data demonstrates that both the number of training objects and their presentation-timing independently affect learning. This independent effect has been obscured by prior literature’s focus on possible interactions between the two. Second, I present a computational model for learning that accounts for both sets of phenomena in a unified way. The Naïve Generalization Model (NGM) offers an explanation of word learning phenomena grounded in category formation. Under the NGM, learning is local and incremental, without the need to perform a global optimization over pre-specified hypotheses. This computational model is tested against human behavior on seven different experimental conditions for word learning, varying over presentation-timing, number, and hierarchical relation between training items. Looking both at qualitative parameter-independent behavior and quantitative parameter-tuned output, these results support the NGM and suggest that rational learning behavior may arise from local, mechanistic processes rather than global statistical inference.

## Introduction

Children famously face ambiguity during morphological and syntactic acquisition [1–5]: how do learners deal with ambiguity when multiple grammars have extensions that are overlapping [6,7]? While words, unlike syntactic units, are often thought of as atomic [8]—cf. [9,10]—a fundamental question in word learning is strikingly similar: how, given only evidence about what objects a word has previously referred to, are children able to generalize to the total class? How does a child end up knowing that “poodle” only picks out a specific subset of dogs despite their overlapping extensions? Learners display remarkable sophistication in their ability to perform statistical inference over the input distribution [11]. Nevertheless, this raises the interesting question of what mechanisms are potentially responsible for actually carrying out such inference. What’s more, learners are sensitive to input conditions that are orthogonal to pure statistical counts (e.g. the timing and relative order of stimuli)—motivating exploration of an architecture that *generates* hypotheses incrementally, prior to their evaluation. The *Immediacy of Linguistic Computation* [12] serves as an informative bottleneck on the acquisition process here: because language (and learning input) unfolds overtime, “if cognitive computations are not made over transient and shifting information as it occurs, they cannot be made at all.” [13] This physical constraint means that learners cannot wait to accumulate evidence and evaluate hypotheses after the fact. Rather, evidence must be accumulated via the hypotheses themselves—an intermediate representation of the data. The learner is limited to evaluating the fit of whatever intermediate representations they posit (be they potential syntactic rules or possible word meanings). One hypothesis will end up winning out because it offers a sufficiently good fit to the data [2], but this does not mean that the final grammar or meaning is globally optimal. In what follows, I first review the literature on generalization in word learning, including a reanalysis of the empirical data related to the effect of stimulus *timing* on learning. I then propose a new computational model of word learning—the Naïve Generalization Model (NGM)—which offers an explicit, mechanistic account of category formation. Unlike global inference approaches, the NGM operates incrementally and locally, and is able to explain both classic and underexplored phenomena such as the suspicious coincidence effect and the presentation-style effect [14–16] within a single framework.

### Word learning and generalization

Language learners need to infer the set of vocabulary items belonging to their particular language based on the patterns of speech produced around them (see [17], among others, for an overview). The lexical entries that learners need to store consist of numerous components. These include a word’s pronunciation, potential syntactic and morphological roles and marking, as well as its meaning [18]. While a substantial literature addresses the problem of resolving referential ambiguity [19–22]—e.g. does “wug” refer to the bird or the squirrel that was being pointed at in the park—the establishment of word meanings does not end there.

Learning even simple categories involves a difficult inductive problem [23]. Consider a sample environment for learning the word “dog”: a child hears an adult speaker point at their pet and refer to it with the label /dOg/. While from the perspective of referential ambiguity the situation is clear—the intended referent is the dog rather than the dishwasher—the space of possible meanings for the phonological label /dOg/ is still quite large. The word may be the particular pet’s name, or it could mean pets generally. It might pick out the set of (all and only) dogs. But it also might select the set of poodles, or mammals, or animals. It might refer to the appearance of the dog: spotted or four-legged or tired. The list goes on. Moving beyond the reference mapping problem for word learning, I focus here on the subsequent and thorny question of meaning generalization. A word’s meaning is better understood as the intended conceptual characterization rather than any particular referent [24–26].

Experimental work on word learning has shown that language learners approach the problem under strong biases with respect to meaning. This is functionally beneficial since it may substantially limit the size of the potential search space. For instance, learners generally assume new words refer to whole objects rather than sets of adjacent parts [27]. There is a bias towards categorization by shape rather than color or size [28]. Prior vocabulary knowledge has a strong effect through mutual exclusivity [29,30]. There are also guiding effects from a host of input signals: syntactic frame [26,31,32], phonetic content [33], social-attentive properties [34,35], etc.

While vocabulary development is clearly affected by such constraints, an enumeration (or even a rich typology) of biases does not explain the underlying mechanisms responsible for learning word meanings. How does this process function? What is the cognitive mechanism behind learners’ remarkable ability to infer the meanings of words based only on a few instances of usage? A helpful conceptualization of this is that words are invitations to form categories [36]. It is striking that infants interpret a word as selecting members of some kind, rather than simply naming an individual referent. Put succinctly by Waxman [37]: “Novel words invite infants to assemble together objects into categories that would otherwise (without linguistic context) be perceived as disparate and distinct.” While category representations do not necessarily *require* explicit linguistic support, experimental evidence supports a tight link between categorization and word learning [38–42].

If hearing a novel word like “fep” can prompt a learner to create a category, we would like to know what knowledge ends up encoded by that process and how. Once a child has seen that “poodle” can refer to whatever instances of poodles they were exposed to, how do they know that “poodle” can refer to all (and only) items in the real class of poodles? This is in contrast to both failing to generalize sufficiently, e.g. erroneously positing that the word only refers to their pet, as well as overgeneralizing that the word selects the set of all dogs.

### Algorithms and rational behavior

One influential account of generalization in word learning is the Bayesian inference theory ([14]—henceforth XT). On this view, learners have some representation of many potential meanings for a word and engage in statistically sensitive calculations to select the hypothesis that is most probable given a distribution of attested exemplars. While a number predictions of a Bayesian inference model are consistent with experimental outcomes [14], these outcomes may not uniquely support the Bayesian view and are open to alternative explanations like I present here. Perhaps the most widely discussed empirical finding in this area is the “suspicious coincidence effect” (SCE)—that an increase in sample size corresponds to the learning of narrower word meanings. However, as discussed below, other empirical findings [15,16] are not easily accounted for by pure statistical inference. In particular, learner behavior additionally depends on the timing of stimulus presentation: whether training items are presented simultaneously (in parallel) or one-by-one (sequentially)—an effect that is consistent and robust over a range of related studies and domains [15,16,43–46]. This experimental manipulation maps well onto real-world conditions: sometimes a learner encounters referents in temporally distributed occurrences rather than grouped together, and one of the goals of this article is to clarify the role and effect of temporal *presentation-style* (PSE) along with SCE that has been obscured by previous literature’s focus on potential statistical interactions [15,16,47].

It is worth noting that the goals of Bayesian approaches to word learning are “at the level of computational theory [48] or rational analysis [49] to understand in functional terms how implicit knowledge and inferential machinery guide people in generalizing from examples rather than to describe precisely the psychological processes involved” [, pg. 250]. However, it does not seem useful—nor do I believe it was Marr’s intention—to treat these levels of analysis as disjoint, without an attempt to match actual human processing behavior. We should aim to connect computational descriptions with *algorithmic* or *mechanistic* explanations of word learning. As noted by Bonowitz *et al*. [50, pg. 60]: “Following the procedures of Bayesian inference by enumerating and testing each possible hypothesis is computationally costly, and so could not be the actual algorithm that learners use... considering the algorithmic level of analysis more seriously can help to address these significant challenges for Bayesian models of cognition.” This is precisely one aim of the NGM.

Word learning involves the construction of mental representations of meaning. While statistical inference accounts of this process posit a global optimization over a (potentially large) set of hypotheses, I instead argue that word learning is an incremental “good enough” process. Like other psycholinguistic processes, this is fundamentally constrained by the Immediacy of Linguistic Computation. From an algorithmic perspective, hypothesized representations are first generated and then only locally revised—as needed—based on input data. On this account, not all plausible hypotheses are simultaneously available. Meanings are built incrementally; any evaluation metric functions only over what is generated from input by the learner. This *Markovian* property is analogous to representations constructed for the perceptual learning of speech [13]: once the learner extracts a belief about what a word potentially means and the original stimulus disappears, then that representation can be updated in the future but the learner is not directly privy to the sequence of input which led to that belief. This kind of limitation is parallel to the divide between *global* and *local* models of referent mapping in word learning [51].

The NGM that I introduce here offers an explanation of word learning phenomena grounded in category formation [52,53]. The NGM outlines a mechanism by which hearing novel words invites a learner to create a new category from component “features” or “properties.” Learners extract properties of objects and store a mental record of them. This is importantly different from statistical inference models of word learning because, under the NGM, word meanings are *generated* by the learner rather than only selected for. Once a representation has been generated for a novel word, the learner may evaluate subsequent labeled objects with respect to this hypothesized meaning; it is these mental representations that serve as the basis of word meanings and generalizations. I term this process “naïve” in the sense that it does not optimize for any particular global value. Under the NGM, both the creation and evaluation of word meanings function locally rather than in terms of a total distribution of input. While the NGM gives rise to *rational* input/output mappings such as SCE, this is the by-product of evaluating such the limited generated hypotheses for sufficient “consistency” with the input rather than complete optimization.

In what follows, I outline previous models, and in particular the Bayesian inference model, along with experimental paradigms, and major phenomena in word meaning generalization. I provide a reanalysis of the experimental data reported in [16] which serves as a replication of the presentation-effect on a large scale while disentangling the main effect of PSE from its lack of interaction with SCE. I then introduce the NGM and its internal mechanisms, as an implementation of the theory of word learning as category formation. The local computation of hypotheses within the NGM can account for both SCE and PSE in a unified way. The NGM simulations are compared with human performance on seven different experimental conditions for word learning, varying over presentation-style, number, and hierarchical relation between training items. Based on two evaluation schemes, one parameter-independent and one parameter-tuned, these results offer support for the NGM over statistical inference models of generalization in word learning, and provide a concrete mechanism for how words invite the creation of categories [36,37].

## Models, experiments, and major findings in generalization

### The allure of optimization

Some of the most popular approaches to generalization in word learning have been built on hypothesis comparison and global optimization (XT and subsequent work): A large set of hypotheses compete based on the relative probability that each hypothesis would be generated by the attested input data. The task is then re-framed as choosing how words map onto those concepts by ruling out impossible or less probable hypotheses until a consistent hypothesis is reached.

The Bayesian model posits that the learner keeps track of their observed sample of referents (out of a known domain of possible items) labeled by a novel word. By assuming that the learner has access to a hypothesis space over the possible concepts that this novel word might map to, the heavy lifting in word learning is understood to arise from a probabilistic model relating individual hypotheses to the observed sample of exemplars. The Bayesian learner evaluates all hypotheses for candidate word meanings according to Bayes’ rule, by computing their posterior probabilities (the likelihood of each hypothesis given the input data *p*(hypothesis|referents)), proportional to the product of prior probabilities *p*(hypothesis) and likelihoods *p*(referents|hypothesis).

This kind of model can be thought of as *global* in two ways [51]. First, calculations of hypothesis-fit to the data are taken over all input received. In the limit, the learner would need to track some record of every attested exemplar in order to compute probabilities over them. Second, all alternative hypotheses are also calculated for goodness-of-fit to the input data. This allows for global comparison, not only between total input and some temporary hypothesis, but between all hypotheses themselves—*contra* the Immediacy of Computation as a bottleneck on learning [12].

This Bayesian inference account makes an intuitive prediction dubbed the “suspicious coincidence effect” (SCE), that if a learner is exposed to some new word “fep” (adapted from [, pg. 249]): “It would be quite surprising to observe only Dalmatians called feps if in fact the word referred to all dogs and if the first four examples were a random sample of feps in the world. This intuition can be captured by a Bayesian inference mechanism that scores alternative hypotheses about a word’s meaning according to how well they predict the observed data, as well as how they fit with the learner’s prior expectations about natural meanings.” Even without explicit computation, it seems natural that a learner should be more likely to notice the uniquely *“poodle”* aspects of some set when shown many poodles to compare, rather than only a single poodle in isolation.

### Xu & Tanenbaum paradigm

One paradigm for investigating learners’ behavior relating to generalization in word learning is a simple labeling task from XT. Unlike in cross-situational paradigms used to probe the referent-mapping problem [20,54], participants are provided with an unambiguous word-label for a set of one or more objects. Given a test-grid of other referents, the experimenter can probe what level of generalization a learner has posited for the novel word-label by measuring subsequent selections from the test-grid.

**Fig 1:**
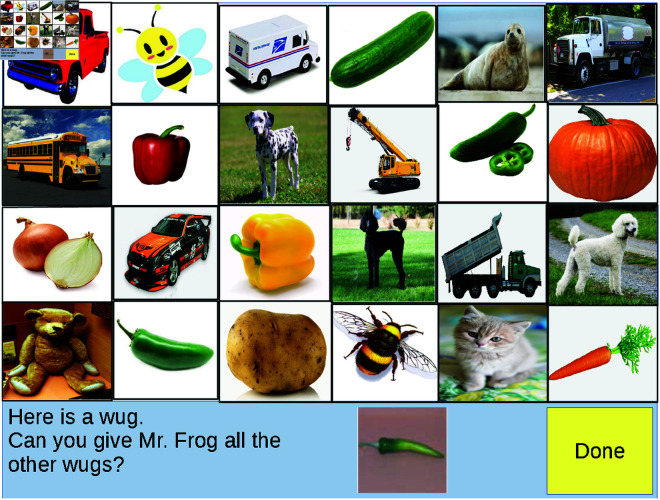
Test-grid from Xu & Tanenbaum. Example word learning trial with test-grid shown to participants. Figure created by the author as an illustrative adaptation of the paradigm in [14–16]; not reproduced from the original.

XT’s experimental paradigm consists of photographs of real objects distributed across three different broad categories or classes (animals, vegetables, and vehicles) to be used as stimuli. The test-grid for these experiments consists of pictures of twenty-four items. This is made up of eight items from each of three classes. For any particular item, it is typical to describe some “basic-level” term [55–57] as the label which would most likely be given to it in isolation (e.g. a dog). In relation to the basic-level term, that same item might also be referred to using a more narrow “subordinate-class” label such as “poodle” or a broader “superordinate-class” label such as “animal.” Within each class in the test-grid, objects exist within three hierarchical levels: two items which come from the same subordinate category (e.g. two jalapeños, or two dalmatians), two items which fit into the same basic-level category as the two “subordinate-level objects” (but which themselves each belong to a distinct subordinate category—e.g. a yellow and red pepper, or a poodle and a golden retriever). Lastly, there are four items which share the same broad class (e.g. animal) but which each belong to distinct basic-level categories (an elephant, a bee, a cat, etc.). The set of “test” objects is consistent across trials with only their position on the grid randomized. A sample of this type of learning trial and test-grid used in XT and subsequent studies [15,16] is shown in Fig 1.

The contextual grounding for this task is that participants are interacting with an alien puppet, ostensibly a monolingual speaker of “alien puppet talk.” On each trial, participants are presented with one or several *training* objects below the test-grid along with an accompanying monosyllabic nonce-word label. For instance, a participant may be shown a picture of a dalmatian with the label “fep” and asked to pick out all the other “feps” for the puppet from the simultaneously displayed test-grid. This paradigm was originally established by XT but has been replicated and extended several times. This includes investigating the effects of distributional structure of stimuli [58] and the role of prior vocabulary knowledge in generalization [59]. These general findings are consistent regardless of whether participants are 3-4 year old children or adults [14] and whether stimuli consist of photos of naturally occurring objects or artificially created patterns [58].

The nature of the timing with which items are presented to learners plays a material role in the outcome of learning as we will see. Adapting terminology from [15] these frameworks are “simultaneous presentation”, in which all training objects are displayed simultaneously directly underneath the test-grid. And “sequential presentation”, in which the training objects are displayed in slightly different locations across very close, yet disjoint times: i.e. the first exemplar is presented at the bottom left for a second and then removed (displaying nothing for a second), then the second exemplar is presented at the bottom middle for a second and removed (again displaying nothing for a second), and then the third exemplar is presented at the bottom right for a second and removed. This sequence is repeated in full two times before the test set is displayed, for a total of six seconds of study time. Once the test set comes up, the training display continues to loop through until the participant has finished that trial. To be clear, the set of exemplars seen by participants in training are held constant between these two conditions of presentation-style. The difference is in the manner in which the training items are encountered—either all at once, or cyclically, one at a time.

### Primary experimental phenomena

When only a single object is presented with a label, then subjects most commonly generalize to the basic-level category, e.g. selecting all *dogs* rather than only *dalmatians* given that the single training item was a dalmatian [14,15]. This is consistent with the robust effects of a basic-level bias [55], although see [60] for the effects of semantic contrast on this comparison. When multiple training examples are presented, then generalization is made narrower (e.g. participants selecting only dalmatians) with respect to the single-exemplar baseline. This “suspicious coincidence effect” (SCE)—that category narrowness is linked to the size of the training sample—has been presented in favor of Bayesian inference in word learning. However this phenomenon is not uniquely consistent with Bayesian inference and is thus open to alternative explanations, such as laid out in this article. Furthermore, the SCE is not the only important factor which influences generalization in word learning. [15] notes that because the implementation in XT assumes inference is performed over an independently sampled distribution of attested referents, the Bayesian account predicts that the order or manner in which training instances are received by the learner should not have an effect on generalization. The likelihoods computed over some sampled set of training objects are agnostic as to the sampling order. XT does mention potential *pragmatic* effects of sampling on representations, *“[a learner may require] a sensitivity to the intentional and epistemic states of the speakers whose communicative interactions produce the examples observed”* (see [61]), but alluding to the idea of pragmatics does not identify a theory or mechanism by which this is actually achieved.

Even when the number and identity of training objects are held constant, the *timing* with which those items are presented to participants has a significant effect on behavior [15,16]. While [16] demonstrate the lack of interference of presentation-style on the SCE (i.e. the *interaction* between presentation-style and training-number on generalization) they do not test for the *main effect* of presentation-style on generalization which is present in their data as well as [15].

A series of experiments in [15] and [16] test two basic presentation frameworks in the same word learning task. Under *simultaneous presentation* all training objects are displayed simultaneously along with the test-grid. This is the setup that XT used to originally measure the SCE: generalization is more narrow when given multiple training items, compared to the single-exemplar baseline. When the same training items are given a single label but displayed to participants in *sequence* rather than all at once, then generalization is significantly broader compared to the simultaneous-presentation baseline, i.e. participants are more likely to select all dogs rather than just dalmatians. [15] argued that presentation-style interacts with, and may explain, the effect of training-number (SCE). [16] found no evidence for an interaction, but they did not analyze or highlight that there is an independent effect of presentation-style. Put simply, both the size of the training set as well as the temporal manner of presentation (PSE) have notable independent effects on the meanings posited by participants. These two phenomena taken together (SCE and PSE) are difficult to explain via a global evaluation model or without taking into account the Immediacy of Computation and the mechanisms of visual comparison that this requires.

While the Bayesian inference model correctly predicts the SCE, the other findings, concerning presentation-style in the data from [15,16], also warrant explanation. There is no mechanism inherent to Bayesian inference which can explain a narrowing of generalizations when subjects are shown objects in short succession as opposed to in parallel. If learners were applying Bayesian inference to maximize the probability of a hypothesized word meaning over global input, then a larger degree of subordinate training items should necessarily correspond to an increased probability of a subordinate word meaning independent of minor timing variations. The Bayesian account also predicts that the SCE should grow as a function of sample size [14]. If it was suspicious to see three dalmatians given a single label, it should be far more suspicious to see twice that many be labeled with the same word. Yet, even when doubling the number of sequential training items from three to six, [15] found no significant difference in generalization. Such findings *“directly contradict the [Bayesian] model’s claim that the likelihood of generalizing at a particular level is scaled exponentially by the number of exemplars at that level”* [15]. This is suggestive that the mechanism underlying the SCE and PSE may not reside in reasoning over distributional statistics, but results from the Immediacy of Computation, and the mechanisms of visual processing and comparative reasoning [15,62] as under the NGM.

## Robustness of the presentation-style effect

The focus of recent literature [15,16,47] has been on explanations of SCE and the factors that do (or do not) influence that effect rather than on the question of word learning or generalization more broadly. It is thus important to clarify some terminology and properly distinguish between the relevant main effects from how they potentially interact. First, as above, the “suspicious-coincidence effect” (SCE) is the effect that when single-item training trials are taken as a baseline, then increasing the number of training items leads to significantly more narrow generalization. Second, the “presentation-style effect” (PSE) is the effect that when the number of training items is held constant, then the presentation-style (timing) with which they are exposed to participants significantly affects generalization. Namely, sequential presentation of training items leads to broader generalization than simultaneous presentation of the same stimuli. Lastly, the “number-timing interaction” measures whether PSE and SCE interact, e.g. if SCE depends on PSE or not.

[16] report the results from a number of variant experiments based on the paradigm from XT and [15]. In addition to the two conditions discussed prior (training-number and presentation-style), another design factor to consider is the order in which participants are exposed to experimental trials. Since training-number is a blocked within-subject manipulation, each participant completes the experiment in one of two possible orders: single-item trials first and multiple-item trials second (1–3) or multiple-item trials first and single-item trials second (3–1). [16] find that, in line with the supplemental experiments reported in [15], *block-order* has a significant effect on learning outcomes.

[16] demonstrate the lack of interference of presentation-style on SCE and present this as a rebuttal to [15], but [16,47] did not test for the *main effect* of presentation-style (PSE). Below, I present an additional analysis of the data from [16]. This analysis replicates the findings reported in [16], but additionally uncovers a robust PSE alongside SCE.

The data from [16] encompass a number of different experiments and the way in which these are analyzed or plotted can obscure crucial patterns. While originally run as twelve separate experiments, a number of manipulations do not have a significant effect on generalization behavior (e.g. same vs. different labels across words, trials grouped by stimulus category or interleaved) and are thus not of primary interest. Here I analyze all the data from [16] together to evaluate the potential manifestations of PSE and SCE. I fit a mixed-effects logistic regression to predict basic-level generalization on each trial using fixed effects of presentation-style, training-number, and block-order (along with their interactions) and random effects for each subject and stimulus class (animals, vegetables, vehicles)—the output of which is summarized in Table 1. When analyzing all trials from [16] (including both first and second-block trials) then there are significant main effects of presentation-style (PSE), training-number (SCE) and block-order, along with a significant three-way interaction (Table 1). The same results hold if generalization is alternatively coded as a gradient outcome (see Supporting Information).

**Table 1:** All trial data from [16].

Predictor	Coefficient	Std. Error	z	p(>|z|)
(Intercept)	–1.610	0.312	–5.155	<.001
Presentation-Style (PSE)	–0.445	0.221	–2.016	0.044
Training-Number (SCE)	1.508	0.129	11.654	<.001
Block-Order	–1.444	0.226	–6.387	<.001
Presentation x Number	0.214	0.246	0.870	.384
Presentation x Block	–0.253	0.440	–0.574	.566
Number x Block	–4.326	0.272	–15.903	<.001
Presentation x Number x Block	1.316	0.494	2.666	.008

**Note:** Dependent variable is the outcome of broad vs. narrow generalization on all trials. Mixed-effects logistic regression predicting generalization based on listed effects as well as random slopes for subject and stimulus class. PSE and SCE emerge as significant main effects along with a three-way interaction between Presentation-Style, Training-Number, and Block-Order.

In order to investigate the shape of the three-way interaction, and in line with the observation from [16] that block-order has a large effect on generalization outcome, I held block-order constant for subsequent analyses. By looking only at the first-block trials (that is the “3” trials in “3–1” ordered experiments and the “1” trials in the “1–3” ordered experiments), we control for this ordering effect and can compare the differing resultant basic-level generalizations between other conditions. For second-block trials I fit a similar mixed-effects logistic regression (see Table 2) which shows no meaningful effects of any training condition. Neither training-number (1 vs. 3 items) nor presentation-style (sequential vs. simultaneous) has a significant effect on generalization during second-block trials. This is very likely a task-effect: once participants are accustomed to the potential hypothesis space and semantic contrasts [60] they generalize only narrow meanings regardless of condition. Adaptation to the generalization paradigm is, perhaps, unsurprising given language users ability to rapidly adapt to systematic cues in other domains such as speech perception (e.g. [13,63] or syntactic processing [64]. Since neither SCE nor PSE manifest on second-block trials (and thus diminish the size of those effects when analyzing all-block trials), I additionally analyzed first-block trials on their own.

**Table 2:** Second-block trial data from [16].

Predictor	Coefficient	Std. Error	z	p(>|z|)
(Intercept)	–9.378	0.705	–13.297	<.001
Presentation-Style (PSE)	–0.220	0.547	–0.403	.687
Training-Number (SCE)	0.009	0.546	0.016	.987
Presentation x Number	–0.207	1.093	–0.190	.849

**Note:** Mixed-effects logistic regression predicting generalization outcome on second-block trials (data from [16]) based on listed effects (Presentation-Style, Training-Number, Number-Timing Interaction) as well as random slopes for each subject and stimulus class. Neither SCE nor PSE manifest on second-block trials.

The same mixed logistic model fit to predict basic-level generalization on first-block trials (see Table 3) shows significant main effects of presentation-style (PSE) and training-number (SCE), with no significant interaction between the two. As with the model fit over all data these trends are robust to gradient vs. discrete coding (see Supporting Information). While the magnitude of PSE is larger in [15] compared to [16], the qualitative trend is consistently replicated between multiple labs and at high power considering the large sample size in [16].

**Table 3:** First-block trial data from [16].

Predictor	Coefficient	Std. Error	z	p(>|z|)
(Intercept)	–0.815	0.417	–1.957	.050
Presentation-Style (PSE)	–1.091	0.410	–2.664	.008
Training-Number (SCE)	4.950	0.680	7.276	<.001
Presentation x Number	0.246	0.792	0.311	.756

**Note:** Mixed-effects logistic regression predicting generalization outcome on first-block trials (data from [16]) based on listed effects (Presentation-Style, Training-Number, Number-Timing Interaction) as well as random slopes for each subject and stimulus class. PSE and SCE emerge as significant main effects.

[16] is correct in noting that *“SCE is robust to presentation timing”* since PSE and SCE do not interact in their data (contra [15] as well as [47]). However the findings from [15] are not moot: presentation-style and training-number nonetheless both have robust and significant independent effects on generalization, motivating a unified explanation from an explicit computation model such as the NGM.

### Presentation-style and learning in other domains

Similar presentation-style effects have been observed in other generalization problems, suggesting that presentation style is a robust phenomenon worthy of explanation. Children are skilled at performing inductive reasoning by means of generalizing a limited piece of evidence about one or a small sample of individuals (e.g. “this peach has a pit”) to an entire category (“peaches have pits,” [44].) In such *property projection* tasks, participants are provided with some fact about a set of objects in the same general category (e.g. “These animals have *type-Z* blood inside”) as training. During testing, participants are asked whether or not they think that same property is also present in some novel object (e.g. “Does this other animal also have type-Z blood inside?”). [65,66] show that even when the training stimuli are held constant, the manner in which they are presented to participants (3 year-olds in this case) induces a significant effect on the outcome. In the Sequential condition, pictures of animals are presented individually, attributed a novel property (“this animal has [property 1/2]”), and placed into a matching pile. In the Simultaneous condition, items are not presented individually. Rather, both samples are presented at the same time in two piles divided by property and described as a total group: “these animals have [property 1],” while “those animals have [property 2].” As in the word learning domain, sequential presentation of exemplars leads to higher rates of broad generalization compared to an otherwise equivalent simultaneously presented set. When learners study two or more instances of the same concept side by side, transfer to more remote instances or acquisition of a new category [67–70] is more likely than when only one instance is studied at a time.

In fact, a wide range of cognitive tasks exhibit a difference in outcome based on the timing or presentation-style of exemplars. In addition to recent findings that the perceptual learning of speech is timing-dependent [13] this includes, but is likely not limited to, inductive category learning [43], visual pattern differentiation [71], the link between conceptual and visual processing [46], visual identification [72], discrimination learning [73], relational reasoning [74], orthographic processing [75], and sensory preconditioning [76], etc. All of these studies show important differences under sequential vs. simultaneous presentation of stimuli. Taken together, these findings on presentation-style seem to reflect more general issues in category learning, such that we should aim for a unified solution.

## Naïve generalization model

Word learning is to construct mental representations of words. In considering the process by which learners encounter input materials and extract potential meanings from that signal (whether in natural settings or experimentally controlled contexts), I would like to highlight a few design principles. This section highlights these properties and introduces the NGM as a formal implementation of the word learning as category formation theory. The full implementation and source code are available open-source on the Open Science Framework as well as via GitHub.

Under the NGM, word learning is a dynamic process whereby potential meanings are built incrementally. The set of potential hypotheses that *might* be derived from a given input is large, but in practice, learners are quick to converge on the correct one most of the time (and only a limited number of active hypotheses are actually considered). I argue that this is due not to the inherent virtue of individual meanings in context, but arises from largely mechanistic means. Statistical trends present in the input may not end up manifesting in cognitive representations depending on properties of the learner (e.g. visual attention) or the learning environment (e.g. presentation-style). Hypothesized representations are generated and only locally revised (as needed) based on input data.

The NGM operates by generalizing semantic representations over “features.” These representations are compared against any subsequent data—also implemented as bundles of features—and potentially updated. This internal representation is consulted whenever the word is heard, and in experimental forced choice tasks like the present paradigm, used to select closely matching objects. I discuss these components of the model in turn. By specifying a mechanism by which mental representations of words are both constructed and evaluated, the NGM is able to capture both supposedly *rational* phenomena such as SCE as well as comparatively arbitrary phenomena like PSE.

### Features

The NGM implementation of “features” follows the classic literature on categories [52,53,77] by representing concepts as salient features/properties. What I call “features” are simply properties that hold for some item [78]. While any two properties may be equally true of an object, in the sense that they are formal operators, it should be clear intuitively that some properties are more salient than others. Consider the number 73. It is probably easier to determine that 73 is odd than it is to determine that 73 is a prime; it is not that its prime-ness is less valid than its being odd, rather it is simply a matter of salience (i.e. how noticeable it is in a particular context).

To simulate the degree to which a property is noticed by a learner, I model two Gaussian distributions over salience. These “salience distributions” differ only in mean; one for features with elevated prominence (here the driving force behind the basic-level bias) and one for all other features. Not to be misconstrued as an explanation of visual processing itself, these salience distributions are akin to an abstract placeholder: a way of formally implementing the notion that some levels of generalization are privileged compared to others. More complete featural or visual theories (see [79,80] for instance) or the shifting distribution of attention based on communicative grounding [81] may offer insight into what drives some level of generalization to manifest as “basic” rather than others, but the NGM captures the way in which categorization and word learning functions to be more narrow or more broad with respect to this baseline condition, regardless of how it is defined. The current implementation is a deliberate simplification aimed at isolating the role of hypothesis generation and evaluation in word learning. The goal is not to model perceptual attention per se, and while incorporating empirically derived measures of perceptual salience may be a valuable direction for future extensions, such detail is orthogonal to the central claims tested here. The main point here is that not all features are created equal.

When a learner encounters a new word, the model samples from the appropriate salience distribution for each feature present. The result is a mental representation as a gradient vector of features (Eq 1). Features are discrete, but learners represent them by assigning and updating probabilities over such feature values. As with any probability values, these mental records are allowed to range on a gradient between zero and one. The upper-bound of one is intuitively important because, conceptually, this corresponds to the feature being as present mentally as it is in the physical world. The learner iterates over the items displayed (if more than one present) and each feature present in the real world will be stored in mental representation at a proportion relative to that feature’s salience.

Such discussion of “features” or properties need not be limited exclusively to low-level perceptual information. Rather, the NGM is able to operate over any kind of conceptual or visual units which may be further built up throughout development. It is the process of word learning to create rich category representations from existing parts. I would stress though that this generation process is local and non-deterministic, so not every possible competitor hypothesis is necessarily activated given a fixed set of stimuli.

To state the process of feature extraction more formally, a vector representation *R* is computed for a label *w* based on an example set of training items *T* by sampling all features *f*_*i*_ in *T* with salience *S*(*f*). This is adapted from classic approaches to category membership calculation [52].

$$R_{w}=\sum_{t\in T}S(f_{i}),\;\;\;0\;\;i\;\;|t_{p}|$$ (1)

*t*_*p*_ is the set of features (or *properties*) of the item *t*. *S*(*f*) is the *salience function* for a feature *f*, which returns a value sampled from the normal distribution with mean μ (dependent on *f*). While features for an object in the world are implemented as formal operators, the mental stored values for a given feature are gradient. The NGM sums the values of each present feature until reaching the ceiling condition (of 1.0). This is in line with previous featural implementations of categories, e.g. [82, pg. 287]kruschke2008models: *“the simplest way [to learn associative strengths] is adding a constant increment to the weight whenever both its source and target node are simultaneously activated.”*

**Fig 2:**
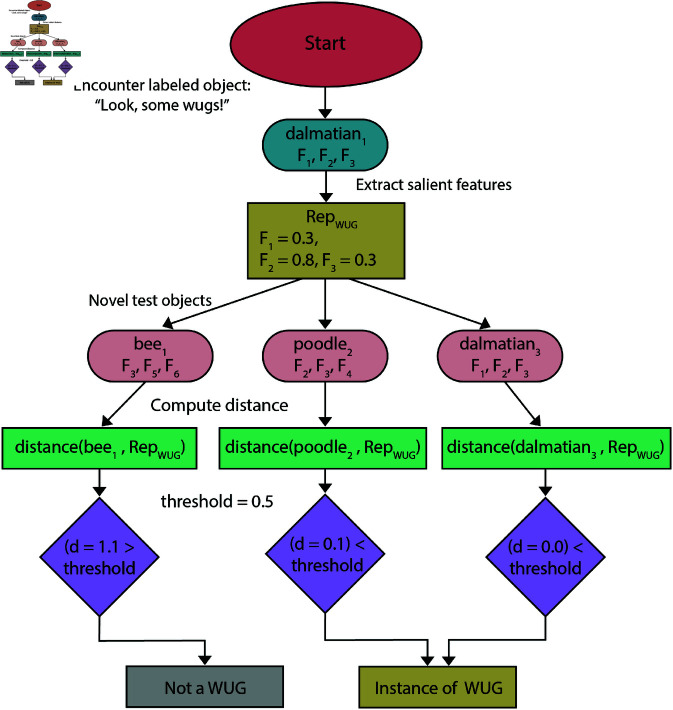
Computation of mental representation from single training example and subsequent comparison to test objects. Exact values are schematic and for illustration only.

The only restriction placed on learning by feature sampling in the NGM is one of consistency. Since some features are necessarily in conflict with one another—no object is both [+round] and [+square]—we would like a learner to not represent both such features within the meaning of a single word. Once a feature has been sampled with salience above a “semantic incompatibility” threshold, any other features which would be in semantic conflict with that are not added to representation. An outline of the feature sampling process and subsequent category determination for novel objects is diagrammed in Fig 2.

### Learning

**Fig 3:**
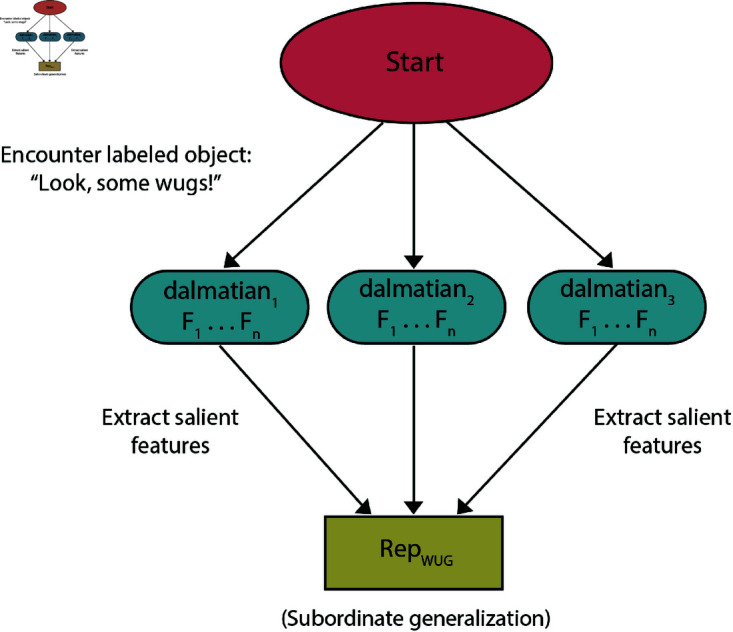
Algorithmic flow chart highlighting a possible path of NGM behavior under parallel-presentation. Typical path of meaning extracted from parallel-presentation trial. All exemplars contribute to initial hypothesized meaning.

When trained on a single exemplar, the experimental finding [14–16] is that learners’ most likely generalization is to the basic-level. This is driven by the privileged status of certain features for generalization over others (and is effected by factors such as the prototypicality of features [83]). When training objects are initially presented simultaneously then a hypothesis category can be formed in a single shot. Thus, when they are co-present, the function which extracts features from a scene is able to compare exemplars directly to exemplars. When features are activated multiple times, they are more likely to be encoded in the category representation [52,82]. The notably rapid ability of people to extract accurate estimates from a set is well-discussed in the literature on ensemble perception [84,85]. Properties which, when encountered in isolation, would not have a significant effect on stored meaning can, through this combination, lead to more narrow generalization. The NGM’s mechanistic account of featural weighting thus makes the same predictions as Bayesian inference with respect to SCE under simultaneous presentation. A typical path from labeling to representation in parallel presentation trials is diagrammed in Fig 3.

**Fig 4:**
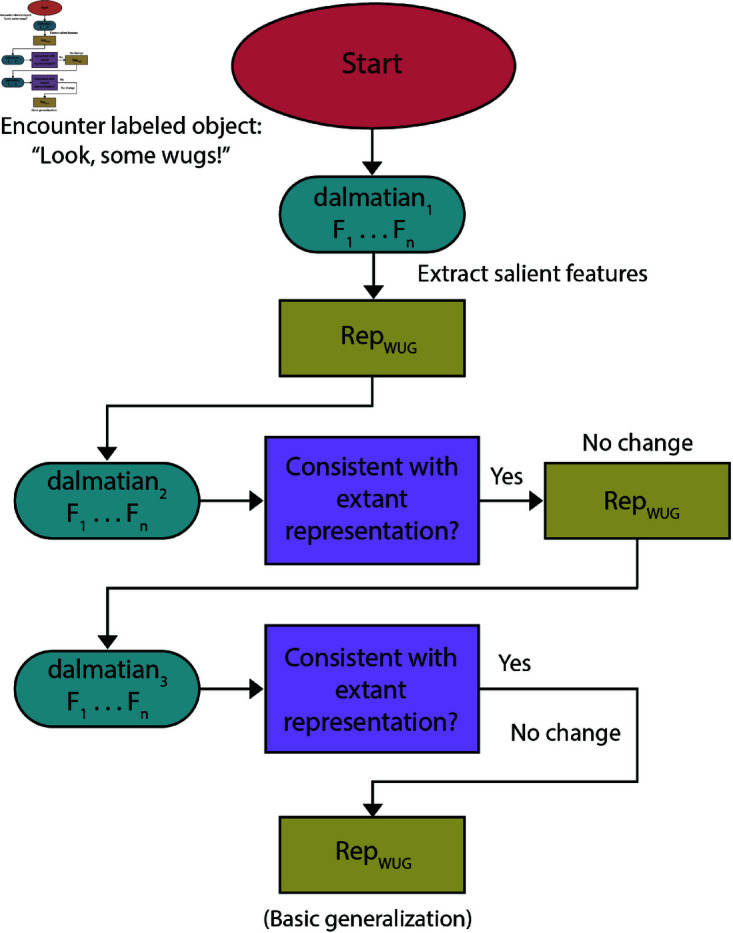
Algorithmic flow chart highlighting a possible path of NGM behavior under sequential-presentation. Typical path of meaning extracted from sequential-presentation trial. Subsequent “training” stimuli do not affect initial hypothesis so long as consistent. If inconsistent, then a new hypothesis is generated (not depicted here).

When the same stimuli are presented in sequence rather than in parallel, learners’ generalizations are more broad [15]. Even though training objects may be shown to learners multiple times, the learner can construct an initial hypothesis only once. After an exemplar has disappeared from view then, due to the Immediacy of Computation, the learner can only refer back to it by consulting some mental representation for the presented word. Once a mental representation exists, there is no onus to change it significantly so long as subsequent objects picked out by the word are congruent with what has been stored. This process is analogous to local models of referent mapping [20,51]. Learners generate a hypothesis and either stick with it if evidence is consistent, or move to a new hypothesis (or otherwise incorporate updates) when faced with inconsistent evidence. When subsequent training instances appear, the original exemplar(s) are no longer present, with only the generated category representation remaining. This means that learners are comparing new exemplars to a category representation rather than directly comparing exemplars with each other. Since all of these trials concern levels of generalization, no new training item will disprove an over-generalized hypothesis. Therefore, learners will simply continue along with whatever initial hypothesis was created. Repeat exposures increase a learner’s *confidence* in the hypothesized meaning rather than triggering any change in the word’s internal contents. This continues until some “convergence point” is reached and a semantic representation is more or less fixed. Such a convergence point is a required component of any model of word learning. The cause of the “basic-level bias” on sequential presentation trials is the same as in the single-exemplar trials: certain types of features lead to privileged levels of generalization. A typical path from labeling to representation in sequential presentation trials is diagrammed in Fig 4. While statistical trends may be latently present in the input distribution, such patterns may remain unnoticed unless they relate back to whatever initial guesses were hypothesized by the learner.

In conceptualizing of word learning as a process of category formation, processing of stimuli is qualitatively different before and after an initial hypothesis representation has been generated. Upon initially encountering referents, when there are no prior hypothesized meanings to compare against, a representation must be created. At future instances of the word’s usage, however, the learner must decide whether this new token is consistent with the current mental representation or not. If the prior hypothesized representation is inconsistent with current input, then an alternative hypothesis is created. When subsequent input is consistent with the prior hypothesis, then the multiple “trials” across sequential presentation do not necessarily impart any change to the internal contents of the word’s represented meaning. Rather it is the learner’s *confidence* in that hypothesis which gets increasingly solidified.

When items are presented in sequence, only salient features are likely to be encoded in representation. This “sparse” representation corresponds to a broad category generalization. Simultaneous presentation, on the other hand, joins all the shared features between presented items [66,70,76]. This combined weighting of otherwise non-salient features leads the representations to correspond to more specific, narrow categories.

### Computing distances

The NGM makes a distance calculation between any new objects and extant mental representations. The comparison of that distance value to a fixed parameter threshold determines category membership. This calculation, adapted from [52], can be written out more mechanically as follows in Eqs 2 and 3:

$$D(r,t)=\sum_{n\in r_{indices}}q(r_{n},t_{n})$$ (2)

$$q(r_{n},t_{n})=\{\begin{array}{ll} r_{n}-t_{n} & \text{if}\;r_{n}\geq t_{n}\\ 0 & \text{if}\;r_{n}<t_{n} \end{array}$$ (3)

**Fig 5:**
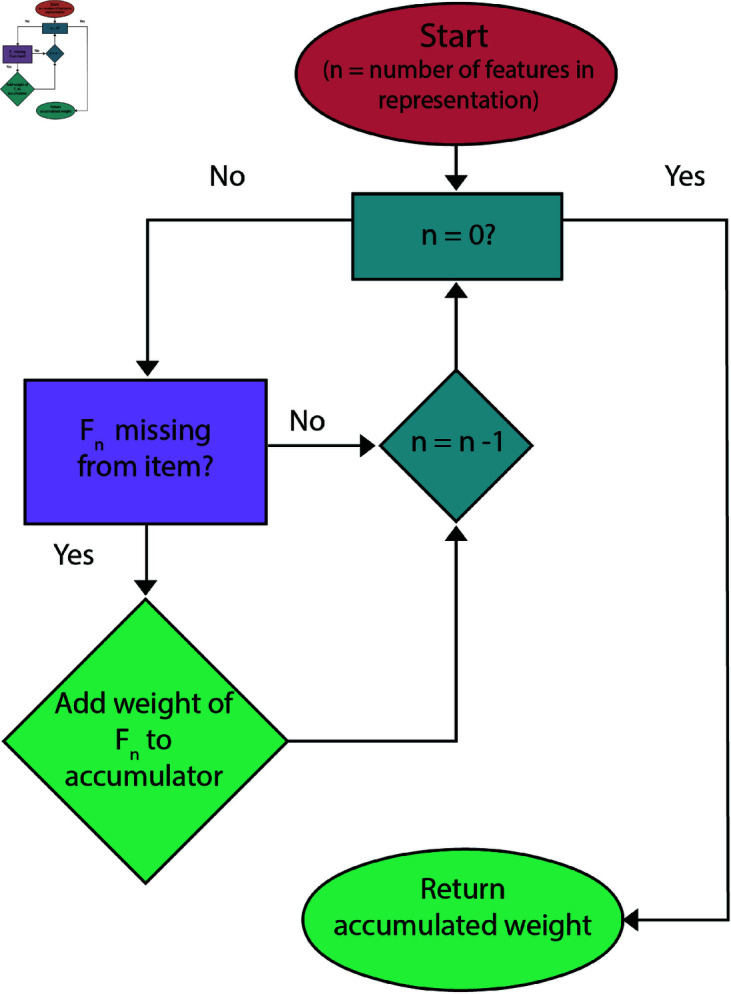
Implementation of distance computation between an object and mental representation under the NGM.

Unpacking Eq 2 a bit, this simply states that to compute a distance between a mental representation *r* and some (test/training) item *t*, the NGM sums up the gap between features in mental representation (*r*_*n*_) and corresponding properties (*t*_*n*_) in the at-issue item. In Eq 3 we see that there is a distance penalty for any feature present in the mental representation *r* that is missing in the test object *t* under consideration. The size of this penalty is the salience of that feature in mental representation. However, there is no cost incurred for features which are present in a test item but are missing in the mental representation of a class. This asymmetry should be intuitive: representations are by definition abstractions, and thus more sparse than actual items (which can be defined under any number of properties). Every object must be perceived as having some color, but that color may play no role in these items’ membership in the of various categories being learned here. See Fig 5 for a schematic of this evaluation function. An example of the full word learning experimental calculation is given in Table 4.

**Table 4:** Example learning and evaluation trial under the NGM.

	Object	Features	Distance
**Training** (“Look, a wug”)	Dalmatian	(A:1, B:1, C:1, D:0)	N/A
**Stored Category**	Representation_WUG_	(A:0.3, B:0.8, C:0.3, D:0)	N/A
**Testing** (“Select the other wugs”)	Bee	(A:0, B:0, C:1, D:0)	1.1
	Dalmatian	(A:1, B:1, C:1, D:0)	**0.0**
	Poodle	(A:0, B:1, C:1, D:0)	**0.3**
	Truck	(A:0, B:0, C:0, D:1)	1.4

**Note:** In this toy example, the initial training set contains a single instance of a dalmatian with features (A:1, B:1, C:1, D:0). From this, the learner extracts a mental representation of (A:0.3, B:0.8, C:0.3, D:0). During testing, a few potential items are all compared against mental representation in order to select category members. Only values present in mental representation but missing from the evaluated items incur a penalty. If the maximum category cutoff were 1.0, then both the dalmatian and the poodle (shown in bold) would be selected in this case.

## Results

The crux of the category generalization problem is that items within each narrow class are hierarchically nested in other, more broad, categories as well. A “poodle” is a “dog” is an “animal.” However, the scoring methodology adopted by previous work on this task [14–16], assesses the matches to a specific level of generalization (subordinate, basic, or super) independently. On the test-grid in such experiments, a given training object will then correspond to two uniquely subordinate-matches, two uniquely basic (non-subordinate) matches, and four uniquely superordinate (non-basic) matches. The mean proportion of generalization to each category is then computed over those totals. For consistency and comparability of evaluation, I implemented the same scoring methodology as previous work [14–16]. Scoring for each level of category generalization is done by, in each trial, dividing the number of objects selected by the participant (or model) by the total number of possible objects for that level in the test-grid. Below I report the results based on two evaluation schemes to compare NGM performance to empirical learning behavior.

### Parameter-independent evaluation

In order to properly evaluate a computational cognitive model, we should note which aspects of the empirical data we deem important for theoretical explanation. The evidence that results from experiments such as [14–16] is informative largely on the basis of indicating which experimental conditions drive a significant difference in participant performance, rather than the exact percentages involved. Whether the magnitude of PSE or SCE is 15% or 30% is of little relevance, since it is the presence of the effect that we are primarily concerned with. Thus just as it is important for a cognitive model to be able to capture precise output given the right parameter values, it is also crucial to determine the degree to which qualitative effects of model performance are driven by factors internal to the model itself or dependent on specific parameter settings.

To investigate the performance of the NGM, I measured the proportion of parameter configurations which result in qualitatively the same trends as human empirical output from [15,16]. This is assessed in two parts, one for SCE and one for PSE. First, following [15], I measured SCE on simultaneous-presentation trials. I defined the SCE as “present” if the proportion of basic-level selections was substantially lower in the multiple-item trials compared with single-exemplar trials. Second, PSE is defined such that sequential presentation results in substantially more basic-level selections compared parallel presentation (while holding constant the number of training items.) “0.15” was chosen as the cutoff for a “substantial” difference for these tests as it is representative of empirical standard deviations in this paradigm. Both required conditions for qualitative evaluation are summarized in Table 5.

**Table 5:** Major patterns to be captured by models of word learning and generalization.

Trial Type	Rate of Basic Generalization	Interpretation
Single Object	Baseline	Basic-level Bias
Simultaneous	At least 0.15 lower than Single	Suspicious Coincidence Effect
Sequential	At least 0.15 greater than Simultaneous	Presentation-style Effect

**Note:** Both the size of the training set (Suspicious Coincidence Effect; SCE) and the temporal manner of presentation (Presentation-style Effect; PSE) exert reliable influences on learner behavior. The “0.15” threshold corresponds to the typical standard deviation in generalization rate reported in [15].

With multiple parameters in the NGM, a large number of configurations are possible for the model to be seeded with. I evaluated 1024 different parameter configurations each run with 1000 simulated “participants.” The output trends of the NGM matched the above criteria for human performance on all runs. The qualitative trends required to be captured by the model are, on the whole, independent of individual parameter setting.

### Parameter-tuned evaluation

**Fig 6:**
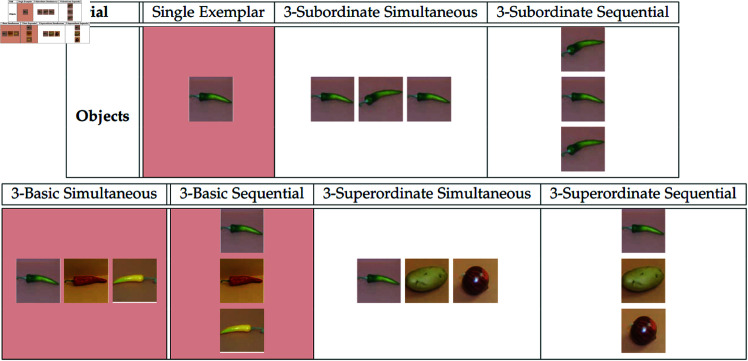
Chart of all seven training configurations. Conditions used for parameter tuning shown in light red. Time during training is indicated within each block vertically; the objects in the parallel condition are co-present at the same time, while the “sequential” trials training objects are never co-present.

Parameter tuning, and subsequent testing, of the NGM was performed by feeding in abstracted versions of the same input data from [15] and scoring the resultant output like the empirical findings. There are seven different trials types (single exemplar trial, three trials with objects presented in parallel, and three trials with objects presented simultaneously) to model, and each experimental condition includes three output proportions (subordinate, basic, and superordinate level generalizations) for a total of twenty-one output means to be compared. To ensure fair evaluation (and avoid over-fitting), I trained the NGM on three of the seven different trial types—training over a single exemplar, training over three basic-level matches in parallel, and training over three basic-level matches in sequence. These conditions are shown in red in Fig 6. Testing was then performed on all experimental conditions from Spencer *et al*. [15] varying the hierarchical organization and presentation-style of the input. Parameter tuning was performed by running a five-way step-wise (*step size* = 0.1) grid search of 1024 configurations (two salience distributions means, salience standard deviation, distance threshold, semantic incompatibility parameter). Parameter tuning here is simply a method for assessing model fit and relative power, and should not be interpreted to reflect any measure of cognitive development (for exact values see Supporting Information).

**Fig 7:**
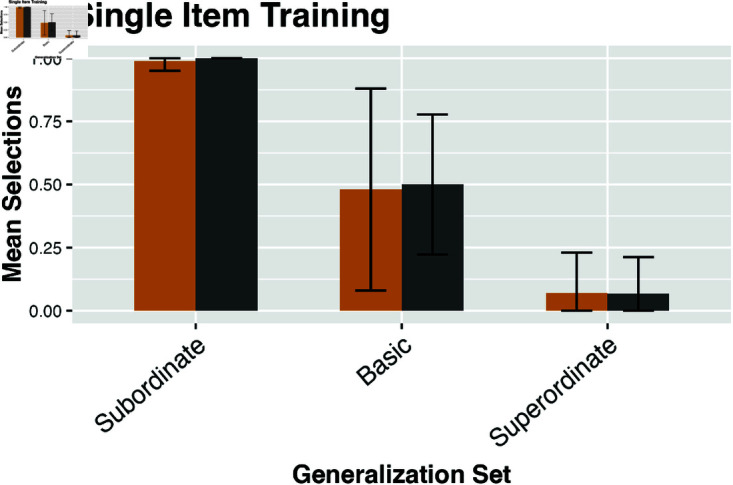
NGM predictions and empirical human data after training on a single item. Gold bars represent human experimental results from Spencer *et al*. [15]; grey bars show output from the NGM. Error bars indicate standard deviations.

**Fig 8:**
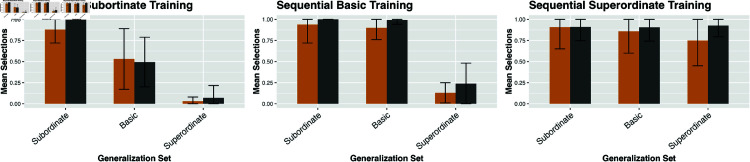
NGM predictions and empirical human data across learning configurations with items presented sequentially. (left) Subordinate training items (e.g., all dalmatians); (center) Basic training items (e.g., all dogs); (right) Superordinate training items (e.g., all animals). Gold bars represent human experimental results from Spencer *et al*. [15]; grey bars show output from the NGM. Error bars indicate standard deviations.

**Fig 9:**
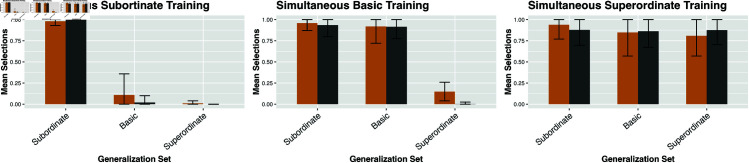
NGM predictions and empirical human data across learning configurations with items presented simultaneously. (left) Subordinate training items (e.g., all dalmatians); (center) Basic training items (e.g., all dogs); (right) Superordinate training items (e.g., all animals). Gold bars represent human experimental results from Spencer *et al*. [15]; grey bars show output from the NGM. Error bars indicate standard deviations.

For each trial, there are three different generalization levels (sub, basic, super), each with a different proportion. To compute the distance from a parameter setting for the model and the empirical data, I summed the absolute value of the difference for the proportion for each level. Each trial configuration was run with 1000 simulated “participants” in the NGM. Even when parameters are fit using only three out of seven of the experimental trial configurations (as above), the empirical fit is very strong as shown in Figs 7, 8 and 9. The mean divergence per trial between the experimental data and the output of the model is 0.049. 96% of trial configurations were within a single standard deviation of the empirical finding.

Overall, the output of the NGM is consistent with human performance on generalization tasks in word learning. The model matches the general empirical trends of note independent of individual parameter values. When tuned on a training set, the output of the NGM has a mean divergence of approximately 0.05 per trial condition compared to the empirical finding. This is within a single standard deviation of human performance 96% of the time. Several general patterns are captured here; the strong basic-level bias in generalization from a single, labeled training instance, the SCE that an increase to training-number leads to narrower generalization, as well as the PSE that sequential-presentation facilitates broader generalization than simultaneous-presentation. All the modeled experimental data was taken from *first-block trials* in order to avoid the effect of block-order uncovered by [16] as well as the supplemental experiments in [15]. While for practical reasons the NGM was evaluated on a set of seven particular experimental conditions, the underlying trends in generalization are robust under numerous related conditions [15,16,65,70].

There are a few cases in which the NGM shows some divergence from the empirical output. The model’s rate of superordinate generalization given a parallel presentation of basic objects is near zero; while the experimental data for such a case is around 0.15. Additionally the model’s rate of generalization to any level given multiple superordinate objects as input is roughly uniform (at approximately 0.85 - 0.92). The empirical pattern is a slight declination from 0.91 subordinate-generalization to 0.75 superordinate-generalization. One potential explanation for this might be in the variance over both individual items as well as by participants. In particular, the range of variation in human participant responses is quite large. The standard deviation for superordinate object training cases varies from a low of 0.17 to nearly 0.3. This is a result both of cross-participant variation as well as item-level differences. Baseline rates of broad generalization are highly variable and governed by the prototypicality of the individual subordinate objects with respect to their containing class [83,86]. In [86] this ranges from a SCE gap of 50% for “dalmatians” being generalized to “dogs” whereas that effect is as low as 10% or even absent in the case of “goldfish” compared to “fish” or “monarch butterflies” compared with “butterflies.” A better grounding of individual item prototypicality and feature salience [79,80] may explain these performance gaps.

## Discussion

Word learning highlights many of the interesting issues in language acquisition. From speech segmentation, to morphological analysis, to referential disambiguation, to the current question of semantic generalization, this is a task which *should* be extremely difficult [23] yet, in general, proceeds smoothly. Much of this success has been attributed to cognitive biases that guide learners toward certain interpretations over others [27,29,30,33,34]. While such biases have been studied extensively, less is known (e.g. [87]) about the mechanism underlying the learning process.

One of the contributions of the Bayesian program to cognitive science is that it *formally implements* a set of computations that can capture intuitions about statistical reasoning and makes related predictions. However, computation is contingent on the representations that have been posited so far. While the Bayesian inference account correctly predicts SCE, it cannot straight-forwardly account for other empirical effects such as PSE. This problem arises because the computations under XT are fundamentally a method for “hypothesis evaluation” without specifying the internal contents of the representations or how they are derived. In the end, it is the combination of representations and the computations performed over them that drive learning. These mental representations are unavoidably derived from the input but may diverge in systematic ways, particularly in light of the Immediacy of Linguistic Computation. Accounts which operate solely at a disjoint computational-level to measure statistical trends will naturally miss some of the puzzle.

The formal NGM model presented in this article offers an explanation of word learning phenomena grounded in category formation [52,53]. As I argue, word learning is fundamentally to construct mental representations of words rather than strictly evaluate them. This does not necessarily maximize global probability of the output vocabulary, but rather the evaluation metric for meanings functions only over what is generated from input by the learner. The NGM explains the mechanisms behind generalization for word learning in a manner that is consistent with and complementary to local models of referent mapping [20,51]. These findings contribute to a more complete picture of word learning.

While the NGM was evaluated over the output of a particular experimental paradigm, the phenomena captured here (SCE and PSE) are a natural function of real-world learning contexts. Sometimes a child will encounter an object in isolation or across time, while in other cases a group of referents is encountered together. This effect of *timing* in particular should not be surprising in light of the Immediacy of Computation. This is not to overlook the limits to the experimental paradigm employed by [14–16]. Since the test-grid was always co-present with at least some training item, it is unclear how long the learning from this paradigm actually persists. In fact, data from [60] indicate that the particular competitors present in the test-grid have a notable impact on learning outcomes. Looking at a variety of inputs that learners receive [88,89] can establish how such paradigms, and the findings based on them, map well onto or deviate from naturalistic contexts. While the NGM is a model for learning word meanings specifically rather than the whole of acquisition, the core tenets of the approach apply broadly. Future work should aim to connect incrementality and the Immediacy of Linguistic Computation to the more general case of the mechanisms used for hypothesis evaluation on more “structural” problems like the acquisition of argument structure [90,91].

While the learning experiments discussed here [14,15] were conducted with English-speaking participants, the underlying principles of hypothesis formation, categorization, and generalization are not language-specific. Any viable theory of word learning should be language-agnostic, in the sense that it posits general cognitive mechanisms that operate across (varied) linguistic contexts. Indeed, recent evidence suggests that children acquiring German, as well as children from a variety of bilingual backgrounds, employ ambiguity resolution strategies—such as mutual exclusivity—in ways consistent with findings from English-learning children [92].

One limitation of the current work is its focus—both in the experimental data and modeling efforts—on concrete noun learning. This is consistent with the large body of research showing that nouns—particularly concrete count nouns with their strong perceptual grounding and clear individuation—are among the earliest acquired, “easiest” words [93,94]. In principle, the NGM applies to any words learnable through observation, including other lexical categories. For instance, many predicates carry the same kinds of hierarchical semantic ambiguity as the object labels under discussion here (e.g. nibble vs. eat vs. consume) and their meanings may be distributed across more abstract dimensions or contexts. Future work is needed to test whether similar generalization dynamics are present beyond the current stimuli and object-generalization paradigm.

Despite these limitations, the NGM provides a concrete mechanism for how words invite the creation of categories [36,37] and makes clear, testable predictions not only about the end state of word learning but also with respect to the path of intermediate representation that children pass through during learning. While the features currently encoded in the model are essentially placeholders, using artificial stimuli or an independent measure of visual salience would generate more precise predictions under the NGM. This connects word learning to well-motivated studies in other domains such as category learning and visual attention [95] and opens up possibilities to look experimentally at how and when learners update their intermediate representations over time. We do after all aim to reach past the *what* of word learning to understand the *how*.

## Conclusion

The NGM offers a cognitively plausible alternative to global statistical inference accounts of word learning. By modeling generalization as a local, incremental process grounded in the immediate construction and evaluation of hypothesized meanings, the NGM captures both classic and underexplored phenomena, including the suspicious coincidence effect and the role of presentation-style. These results suggest that learners may not be globally optimizing over the input, but rather, constructing meanings on the fly—using only stored representations along with the available perceptual input to generate “good enough” hypotheses for word meanings. This framework not only aligns with known processing constraints but also provides a mechanism by which novel words can meaningfully invite the formation of new categories.

## Supporting information

S1 Appendix Parameter settings and gradient analysis of PSE in Lewis and Frank (2018) (PDF)
