# Supplementary material for: Word learning as category formation
Source: PLoS One. 2025 Jul 3;20(7):e0327615. doi: 10.1371/journal.pone.0327615 (PMC12225872; doi:10.1371/journal.pone.0327615)
Supplement: S1 Appendix — (PDF) [file pone.0327615.s001.pdf]

S1 Appendix. Parameter Settings and Gradient Analysis of PSE in Lewis and Frank (2018)

Spencer Caplan

Tuned Parameters

Table A1. Tuned parameter values used in simulations.

**Parameter**	**Tuned Value**
Prominent salience mean	0.5
Non-prominent salience mean	0.4
Salience standard deviation	0.25
Distance threshold	0.4
Semantic incompatibility parameter	0.1

Gradient Analysis of PSE in Lewis and Frank (2018)

The main analysis for generalization-level was scored using the binary outcome of broad (basic) vs. narrow (subordinate) meanings. This excluded the minority of trials on which a participant selected some, but not all, of the basic-level matches. To make sure that my analyses of SCE and PSE are not being disrupted by removing these potentially "uncertain" participants, I additionally ran analyses over the whole set of trials (including the 6.7%, 104 out of 1560, of trials with mixed test selections). Generalization level outcomes were coded as a gradient measure and fit with mixed-effects linear regressions. This alternative coding scheme did not have a significant effect on the presence of either SCE or PSE (See Table A2 for all trials) nor on the shape of the three-way interaction between Presentation-Style, Training-Number, and Block-Order (see Table A3 for second-block trials and Table A4 for first-block trials).

Table A2. Regression results using all trials from Lewis and Frank (2018). Dependent variable is the generalization-level outcome on all trials. Linear mixed model predicting generalization based on listed effects as well as random slopes for subject and stimulus class. PSE and SCE emerge as significant main effects along with a three-way interaction between Presentation-Style, Training-Number, and Block-Order.

**Predictor**	**Coefficient**	**Std. Error**	**z**	**p-value**
(Intercept)	0.303	0.043	7.073	.015
Presentation-Style (PSE)	-0.048	0.023	-2.113	.035
Training-Number (SCE)	0.197	0.012	16.414	<.001
Block-Order	-0.181	0.023	-8.051	<.001
Presentation × Number (NTI)	0.020	0.024	0.833	.405
Presentation × Block	-0.015	0.045	-0.336	.737
Number × Block	-0.535	0.024	-22.242	<.001
Presentation × Number × Block	0.161	0.048	3.339	<.001

Table A3. Regression results for second-block trials only. Dependent variable is the outcome of broad vs. narrow generalization proportion. Linear mixed model predicting generalization based on presentation-style, training-number, the presentation-number interaction, as well as random slopes for subject and stimulus class. Neither SCE nor PSE manifest on second-block trials.

**Predictor**	**Coefficient**	**Std. Error**	**z**	**p-value**
(Intercept)	0.170	0.044	3.827	.052
Presentation-Style (PSE)	-0.007	0.025	-0.298	.766
Training-Number (SCE)	0.016	0.025	0.647	.518
Presentation × Number (NTI)	0.005	0.050	0.099	.921

Table A4. Regression results for first-block trials only. Linear mixed model predicting generalization based on presentation-style, training-number, the presentation-number interaction, as well as random slopes for subject and stimulus class. PSE and SCE emerge as significant main effects.

**Predictor**	**Coefficient**	**Std. Error**	**z**	**p-value**
(Intercept)	0.437	0.043	10.121	.004
Presentation-Style (PSE)	-0.088	0.032	-2.769	.006
Training-Number (SCE)	0.379	0.032	11.948	<.001
Presentation × Number (NTI)	0.035	0.063	0.554	.579
